# Elevated endotoxin levels in non-alcoholic fatty liver disease

**DOI:** 10.1186/1476-9255-7-15

**Published:** 2010-03-30

**Authors:** Alison L Harte, Nancy F da Silva, Steven J Creely, Kirsty C McGee, Thomas Billyard, Elham M Youssef-Elabd, Gyanendra Tripathi, Esmat Ashour, Mohga S Abdalla, Hayat M Sharada, Ashraf I Amin, Alastair D Burt, Sudhesh Kumar, Christopher P Day, Philip G McTernan

**Affiliations:** 1 University of Warwick, Unit for Diabetes and Metabolism, Warwick Medical School, Clinical Sciences Research Institute, UHCW, Clifford Bridge Road, Coventry, CV2 2DX, UK; 2 Biochemistry Dept, National Research Center, Dokki, Giza, Egypt; 3 Chemistry Dept, Faculty of Science, Helwan University, Egypt; 4 Clinical Pathology Dept, National Institute of Diabetes & Endocrinology, Cairo, Egypt; 5 School of Clinical Medicine (Hepatology), Floor 4, William Leech Building, The Medical School, Framlington Place, Newcastle upon Tyne NE2 4HH, UK

## Abstract

**Background:**

Emerging data indicate that gut-derived endotoxin may contribute to low-grade systemic inflammation in insulin resistant states. This study aimed to examine the importance of serum endotoxin and inflammatory markers in non-alcoholic fatty liver disease (NAFLD) patients, with and without type 2 diabetes mellitus (T2DM), and to explore the effect of treatment with a lipase inhibitor, Orlistat, on their inflammatory status.

**Methods:**

Fasted serum from 155 patients with biopsy proven NAFLD and 23 control subjects were analysed for endotoxin, soluble CD14 (sCD14), soluble tumour necrosis factor receptor II (sTNFRII) and various metabolic parameters. A subgroup of NAFLD patients were re-assessed 6 and 12 months after treatment with diet alone (n = 6) or diet plus Orlistat (n = 8).

**Results:**

Endotoxin levels were significantly higher in patients with NAFLD compared with controls (NAFLD: 10.6(7.8, 14.8) EU/mL; controls: 3.9(3.2, 5.2) EU/mL, p < 0.001); NAFLD alone produced comparable endotoxin levels to T2DM (NAFLD: T2DM: 10.6(5.6, 14.2) EU/mL; non-diabetic: 10.6(8.5, 15.2) EU/mL), whilst a significant correlation between insulin resistance and serum endotoxin was observed (r = 0.27, p = 0.008). Both sCD14 (p < 0.01) and sTNFRII (p < 0.001) increased with severity of fibrosis. A positive correlation was also noted between sTNFRII and sCD14 in the NAFLD subjects (r = 0.29, p = 0.004).

Sub-cohort treatment with Orlistat in patients with NAFLD showed significant decreases in ALT (p = 0.006), weight (p = 0.005) and endotoxin (p = 0.004) compared with the NAFLD, non-Orlistat treated control cohort at 6 and 12 months post therapy, respectively.

**Conclusions:**

Endotoxin levels were considerably increased in NAFLD patients, with marked increases noted in early stage fibrosis compared with controls. These results suggest elevated endotoxin may serve as an early indicator of potential liver damage, perhaps negating the need for invasive liver biopsy. As endotoxin may promote insulin resistance and inflammation, interventions aimed at reducing endotoxin levels in NAFLD patients may prove beneficial in reducing inflammatory burden.

## Background

Non-alcoholic fatty liver disease (NAFLD) is a condition in which triglycerides accumulate within the hepatocytes of patients with only moderate intake of alcohol or none at all. NAFLD affects 20-30% of the general Western population [1], and the condition is strongly associated with insulin resistant states such as obesity [2], metabolic syndrome [3] and type 2 diabetes mellitus (T2DM) [4]. In most cases the liver pathology is non-progressive, however some patients will develop non-alcoholic steatohepatitis (NASH) and fibrosis, which may progress to liver cirrhosis over time [1].

NAFLD and other insulin resistant states are associated with activation of the innate immune system resulting in chronic sub-clinical inflammation, particularly affecting the adipose tissue [5,6]. However, the underlying mechanisms behind this association remain poorly understood. In recent years, the major outer membrane constituent of gram-negative bacteria, lipopolysaccharide (LPS), also referred to as endotoxin, has been implicated as potentially important in this regard - as it is a potent inducer of inflammation. It activates the innate immune pathway via stimulation of toll-like receptors (TLRs), enabling a rapid reaction to infection, and represents the first line of defence against gram-negative infections [7]. TLRs combine with the pattern recognition molecule CD14 to form a complex (TLR4-CD14), which activates the NFκB pathway, thus sCD14 levels are strongly associated with endotoxin levels. This, in turn, induces the expression of inflammatory mediators (adipocytokines) such as leptin, tumour necrosis factor-α (TNFα) and interleukin-6 (IL-6), amongst others [7,8]. As a result, an acute phase response [8] is initiated, in conjunction with the liver, as the latter is the primary site of endotoxin clearance under typical physiological conditions.

In normal circumstances, only small amounts of endotoxin will cross from the intestinal lumen into the systemic circulation and the absorbed endotoxin will rapidly be removed by monocytes, particularly resident kupffer cells within the liver. However, emerging evidence indicates that chronic, low level elevation of endotoxin levels may play a role in insulin resistant states. Elevated endotoxin levels have been noted as an aggravating factor in alcoholic liver disease [9], whilst Erridge and colleagues observed that a high-fat meal induces post-prandial low grade endotoxinaemia [10]. In addition, recent studies by Ghoshal and colleagues identified a mechanism through which long chain dietary fats promote the transport of gut-derived LPS into the bloodstream [11]. Furthermore, studies have confirmed that intestinal permeability and small intestinal bacterial overgrowth are increased in NAFLD patients and that these factors are associated with the severity of hepatic steatosis [12]. Indeed, our previous studies in human adipose tissue have shown that both states of obesity and T2DM induce up-regulation of TLRs [13], whilst treatment of human subcutaneous adipocytes with endotoxin leads to activation of the NFκB pathway and subsequent downstream secretion of TNFα and IL-6 [13]. With chronic low grade endotoxinaemia also identified within mouse models of obesity/diabetes [14], as well as NASH [15], many studies support a possible role for endotoxin in metabolic disease.

For our present studies, we hypothesised that endotoxin levels are increased in patients with NAFLD. Therefore, we 1) examined levels of circulating endotoxin in a large cohort of patients with NAFLD in comparison with healthy, control subjects; conducting further sub-analysis to determine differences in endotoxin levels in NAFLD and NASH patients 2) assessed whether endotoxin levels correlate with disease severity and with markers of inflammation and insulin resistance and 3) explored whether treatment with Orlistat, a lipase inhibitor used as a weight-reducing agent, is associated with a reduction in endotoxin levels.

## Materials and methods

### Subjects

Fasted human blood was collected from a total of 155 patients (50 ± 12 years, 69 males) with biopsy proven NAFLD and 23 healthy controls (45 ± 10 years, 8 males). The sub-categories of NAFLD were determined by liver biopsies and liver function tests, by ballooning and/or fibrosis in accordance with the proposals set out by Brunt *et al *[16]. Diabetic status was also ascertained by glucose and insulin levels. The subjects were firstly divided into 2 categories: simple fatty liver disease (NAFLD, n = 63) and steatohepatosis (NASH, n = 92) in order to determine differences between the 'non-progressive' NAFLD and NASH, which has pathogenic implications. These cohorts were then further subdivided into fibrosis and cirrhosis (n = 20) and T2DM (DB, n = 49) to investigate the influence of these disease states on endotoxin and inflammatory mediator levels. In a subgroup of 14 non-diabetic patients with NAFLD, anthropometric and biochemical parameters (BMI, insulin, glucose, lipid profile, C-peptide and ALT) were assessed at baseline and 6 and 12 months post treatment with Orlistat (120 mg twice daily, n = 8) or a placebo (n = 6), as part of a randomised trial. The study was approved by the Local Ethics Research Committee and informed consent was obtained from all participants.

### Biochemical analyses

In patients with NAFLD, serum levels of lipids, glucose, ALT and insulin were measured consecutively in the hospital's laboratory. The method for measuring insulin was the same throughout the study period via routine biochemistry lab protocols. In control subjects, insulin measurements were performed by a solid phase enzyme amplified sensitivity immunoassay (Linco Research, St Charles, MO), and glucose was measured by a glucose oxidase method (YSL 200 STAT plus). Homeostasis model assessment for insulin resistance (HOMA-IR) was calculated for all patients using the HOMA formula: HOMA-IR = Fasting insulin (mU/L) × plasma glucose (mmol/L)/22.5.

### Analysis of circulating endotoxin levels

Serum endotoxin was analysed using a commercially available QCL-1000 LAL Endpoint Assay (Lonza, New Jersey, USA). The assay, and the values given by the manufacturer for intra-assay CV (3.9 ± 0.46) and inter-assay CV (9.6 ± 0.75), have been validated in our laboratory, as detailed previously [12].

### Assessment of inflammatory markers

Sera were analysed by enzyme-linked immunosorbent assay (ELISA) for quantification of the inflammatory markers, soluble CD14 (sCD14) and soluble tumour necrosis factor (TNF)-α receptor II (sTNFRII) (R&D Systems, UK). According to the manufacturers', intra- and inter-assay coefficients of variation were < 7% for all assays.

### Statistical analysis

Statistical analysis was carried out using SPSS 16.0 for Windows software (SPSS Inc, Chicago, IL). Variables were expressed as mean ± standard deviation (SD) or median (interquartile range), depending on assessment for Gaussian distribution. Data were analysed by parametric or non-parametric tests, accordingly. Multivariate linear regression analyses were used to explore the effects of T2DM and fibrosis stage on levels of sCD14. Probability values (two-sided) were considered significant at p < 0.05.

## Results

### Serum levels of endotoxin

**Figure 1:**
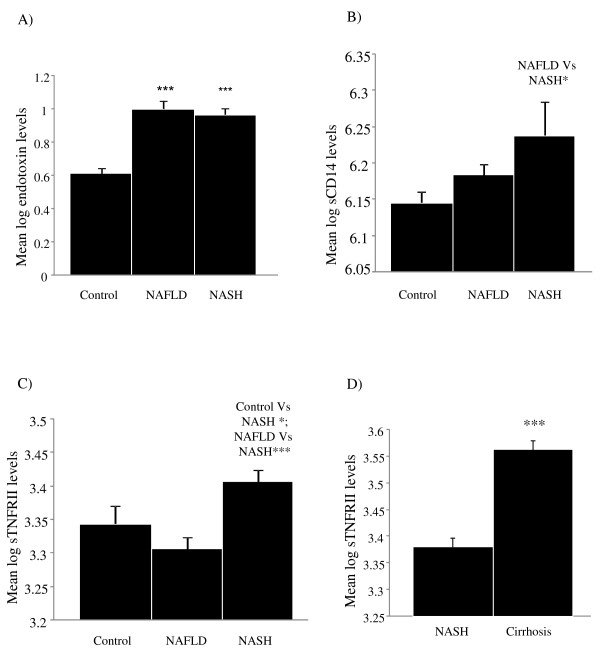
**Levels of endotoxin, sCD14 and TNFRII in NAFLD and NASH subjects compared with controls**. The figures show the mean log endotoxin levels (A), mean log sCD14 levels (B) and mean log TNFRII levels (C) in control, NAFLD and NASH subjects for the whole cohort. The last figure (D) shows the mean log of TNFRII levels in NASH versus cirrhosis (* p < 0.05, ** p < 0.01, ***p < 0.001). Endotoxin or sCD14 showed no significant differences between NASH and cirrhosis in these subjects (data not shown).

**Figure 2:**
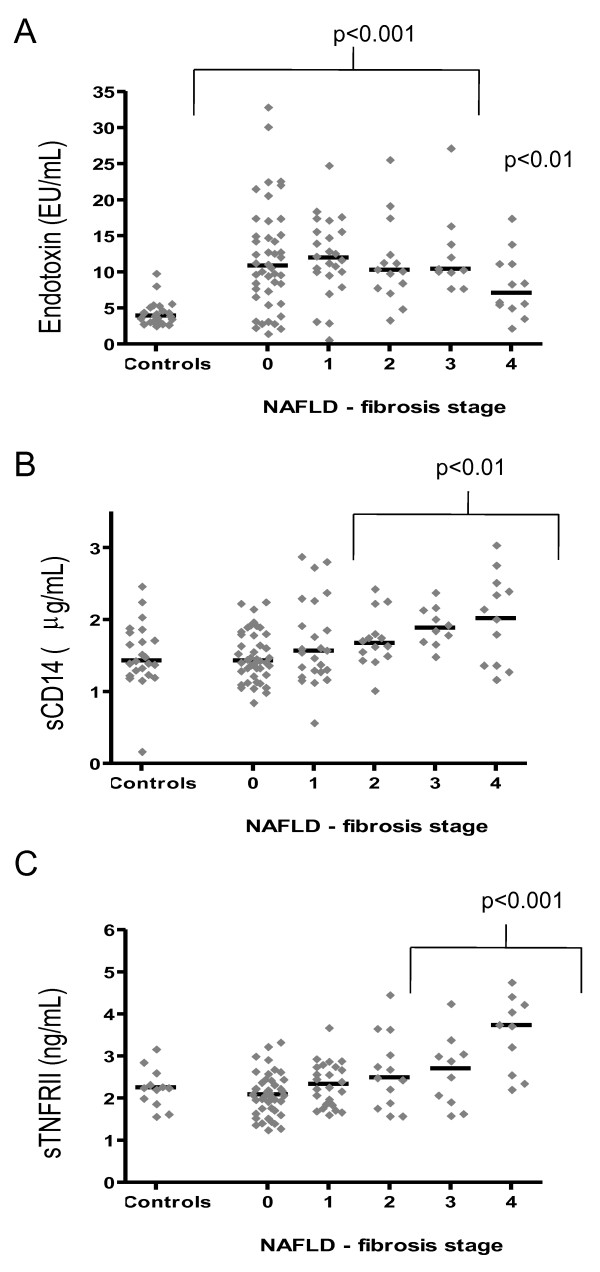
**Serum levels of endotoxin (A), sCD14 (B) and sTNFRII (C) in 23 healthy controls and 155 patients with NAFLD**. The horizontal lines represent the median of the data. Statistical analysis compared the log mean serum levels of the inflammatory markers at each fibrosis stage of liver disease against the log mean serum levels of healthy controls subjects, (p < 0.01, p < 0.001).

**Figure 3:**
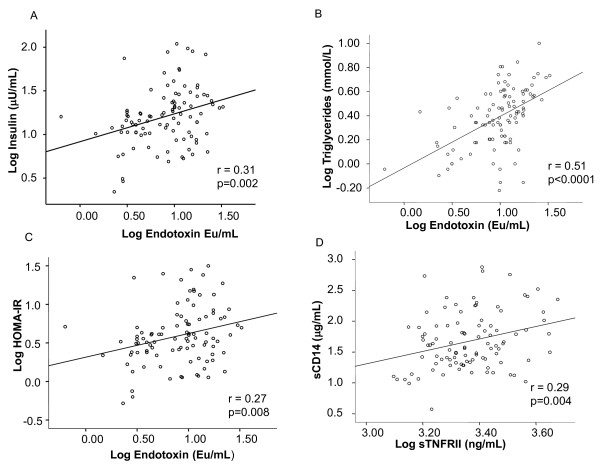
**Correlations between log endotoxin and log fasting insulin, log triglycerides, HOMA-IR, and between sTNFRII and sCD14**. The figures show Pearson correlations between log endotoxin (EU/mL) and log fasting insulin (μU/mL) in the whole cohort (A) log triglycerides (mmol/L) in patients with NAFLD (B) log serum levels of endotoxin and HOMA-IR in the whole cohort (C). The lines of best fit are also shown: a) r = 0.31, p = 0.02, b) r = 0.51, p < 0.0001 c) r = 0.27, p = 0.008. A Pearson correlation between log sTNFRII (ng/mL) and sCD14 (μg/mL) in patients with NAFLD is also shown (D). The line of best fit is: d) r = 0.29, p = 0.004.

Serum endotoxin levels were significantly higher in patients with NAFLD and NASH compared with healthy controls, independent of diabetic status (p < 0.001, Table 1, Figure 1A), whilst no significant difference between endotoxin levels in NAFLD, NASH and cirrhosis subjects was observed (Figure 1A, Table 2, cirrhosis data not shown). Further sub-analysis showed endotoxin levels were comparable, independent of fibrosis score (1-3) except for stage 4, in which endotoxin levels were significantly lower compared with stage 3 (endotoxin, fibrosis score 0: 11.9 ± 1.1 EU/mL, 1: 12.1 ± 1.1 EU/mL, 2: 11.4 ± 1.6 EU/mL, 3: 12.6 ± 1.8 EU/mL and 4: 8.2 ± 1.3 EU/mL, p = 0.03 Figure 2A). Endotoxin levels correlated strongly with insulin levels in the whole cohort (r = 0.31, p = 0.002, Figure 3A), fasting triglycerides in patients with NAFLD (r = 0.51, p < 0.0001, Figure 3B) and with HOMA-IR levels in the whole cohort (r = 0.27, p = 0.008, Figure 3C).

**Table 1:** Clinical and biochemical characteristics of NAFLD Compared with Control Subjects.

	*NAFLD*	*Controls*	*P-value*
**Age** **(yrs)**	49.1 ± 12.6	44.6 ± 9.9	NS

**BMI** **(kg/m^2^)**	34.0 ± 6.0	26.4 ± 4.5	0.0001

**Endotoxin #:** **(EU/mL)**	10.6(7.8, 14.8)	3.9(3.2, 5.2)	0.0001
**Fatty Liver #**	11.7(7.3, 15.6)	n/a	0.0001
**NASH #**	10.5(8.0, 14.0)	n/a	0.0001
**Cirrhosis #**	7.1(5.2, 11.2)	n/a	0.01

**Insulin **# **(μU/mL)**	18.5(10.3, 28.8)	12.9(10.6, 16.0)	0.001

**Glucose **# **(mmol/L)**	5.5(4.8, 6.5)	5.3(4.9, 6.2)	NS

**HOMA-IR **#	4.2(2.5, 7.5)	3.1(2.4, 4.1)	0.001

**TNF-α **# **(pg/mL)**	5.8(4.5, 8.1)	11.2(9.5, 12.2)	0.0001

**sCD14 **# **(ng/mL)**	1623(1370, 2013)	1431.3 ± (1244, 1827)	NS

**sTNFRII **# **(pg/mL)**	2229.2(1865.6, 2879.9)	2253.2(1900.4, 2536.5)	NS

Data are presented as mean (± SD) unless log transformed (#) in which case they are presented as mean (interquartile range).

**Table 2:** Clinical and biochemical characteristics of NAFLD Compared with NASH Subjects.

	NAFLD	NASH	*P*-value NAFLD Vs NASH
**Age (yrs)**	47.2 ± 11.1	50.4 ± 13.3	NS

**BMI (kg/m^2^)**	32.6 ± 5.7	35.0 ± 6.0	p < 0.05

**Endotoxin (EU/mL) #**	12.3(7.3, 15.6)	10.9(7.8, 13.9)	NS
**Fibrosis score: 0**	12.3(6.6, 17.2)	8.6(5.5, 11.7)	
**1**	13.3(11.1, 15.6)	11.7(8.0, 17.2)	
**2**	7.1(7.1, 7.1)	11.7(8.2, 15.0)	
**3**	NA	12.6(9.4, 14.5)	
**4**	NA	8.2(5.2, 11.2)	

**Insulin (μU/mL) **#	18.3(8.1, 22.3)	30.4(14.6, 36.3)	p < 0.001
**Fibrosis score: 0**	16.7(8.1, 21.1)	29.1(9.8, 26.2)	
**1**	37.2(8.7, 76.3)	26.8(14.2, 33.4)	
**2**	NA	27.4(16.3, 36.9)	
**3**	NA	38.6(18.4, 67.2)	
**4**	NA	37.0(17.6, 54.8)	

**Glucose (mmol/L) **#	5.5(4.8, 6.1)	6.6(4.8, 7.0)	p = 0.01
**Fibrosis score: 0**	5.4(4.8, 5.9)	6.9(4.5, 6.2)	
**1**	6.6(4.8, 8.3)	5.5(4.7, 5.8)	
**2**	6.7(6.7, 6.7)	5.7(4.5, 6.4)	
**3**	NA	8.0(5.5, 9.2)	
**4**	NA	8.2(5.0, 10.8)	

**HOMA-IR **#	4.8(1.7, 5.6)	8.6(3.3, 13.3)	p < 0.001
**Fibrosis score: 0**	4.1(1.7, 5.4)	7.4(1.9, 7.3)	
**1**	12.4(1.8, 27.0)	7.0(3.0, 8.6)	
**2**	NA	6.9(3.3, 9.1)	
**3**	NA	12.8(4.4, 22.5)	
**4**	NA	12.7(7.0, 21.9)	

**TNF-α (pg/mL) #**	8.3(4.2, 7.3)	14.7(5.0, 8.9)	p < 0.05
**Fibrosis score: 0**	8.6(4.2, 7.4)	7.4(4.7, 8.0)	
**1**	6.0(4.0, 8.9)	16.6(5.1, 6.9)	
**2**	6.3(6.3, 6.3)	8.5(4.9, 10.6)	
**3**	NA	43.7(4.4, 21.6)	
**4**	NA	6.7(4.7, 9.0)	

**sCD14 (ng/mL) #**	1575.0(1242.6, 1840.8)	1805.2(1374.8, 2169.6)	p = 0.01
**Fibrosis score: 0**	1533.3(1224.6, 1809.2)	1716.0(1331.1, 2018.2)	
**1**	1851.2(1319.3, 2408.3	1642.2(1302.1, 1857.5)	
**2**	1747.1(1747.1, 1747.1)	1829.3(1558.2, 2253.3)	
**3**	NA	1870.0(1680.2, 2080.5)	
**4**	NA	2000.3(1361.0, 2460.1)	

**sTNFRII (pg/mL) #**	2097.3(1642.42453.7)	2676.9(2075.6, 3034.2)	p < 0.001
**Fibrosis score: 0**	2062.9(1621.2, 2445.0)	2491.0(2076.8, 2377.6)	
**1**	2292.8(1729.0, 3067.5)	2330.2(1991.2, 2752.2)	
**2**	2494.9(2494.9, 2494.9)	2597.2(1833.7, 3341.7)	
**3**	NA	2688.9(1962.0, 3047.1)	
**4**	NA	3538.0(2558.3, 4229.2)	

Data are presented as mean (± SD) unless log transformed (#) in which case they are presented as mean (interquartile range). NA refers to no data available as there were no subjects in this group.

### Serum levels of sCD14

To further assess the potential role of endotoxin in NAFLD, we also measured serum levels of sCD14, as elevated levels of this protein may reflect increased endotoxin activity *in vivo *[8]. No significant differences in sCD14 levels were noted upon analysis of control versus NAFLD and NASH subjects. However, NASH subjects did show significantly higher levels of sCD14 than NAFLD subjects (p = 0.01, Figure 1B, Table 2). Analysis of fibrotic stage showed that sCD14 levels increased with severity of fibrosis, with significant differences observed at stages 2-4 (p < 0.01, Figure 2B). Lastly, an association between sCD14 and sTNFRII levels was reflected in the significant, positive correlation present between these two variables in the NAFLD cohort (r = 0.29, p = 0.004, Figure 3D), a finding that was absent in the healthy control cohort (data not shown).

### Serum levels of sTNFRII

To assess a potential relationship between endotoxin levels and inflammation, we also measured serum levels of sTNFRII. TNFRs are released from the cell surface of monocytes as a result of the same inflammatory mediators that are known to induce TNFα [17]. However, due to the short half-life of TNFα, TNFRs are considered to be a more accurate reflection of TNFα activity. For sTNFRII, the results show that serum levels were significantly higher in patients with NASH compared with healthy control and NAFLD subjects, respectively, independent of diabetic status (Figure 1C, Table 2, diabetic data not shown). Further sub-analysis showed sTNFRII levels were significantly elevated in subjects with cirrhosis compared with those subjects with NASH (Figure 2D). In a similar pattern to that of sCD14, sTNFRII was significantly elevated with increased fibrosis (stages 3 & 4) compared with controls (p < 0.001, Figure 2C).

### The effect of diabetic status on levels of endotoxin and sCD14

Endotoxin levels were similar at all stages of NAFLD, independent of diabetic status (p = 0.049). In contrast, serum levels of sCD14 were significantly higher in NASH patients with T2DM compared with control and NAFLD subjects with and without T2DM (p < 0.01). This difference, however, could be due to the stage of liver disease, as noted by an increased frequency of advanced fibrosis (i.e. bridging fibrosis or cirrhosis) in T2DM patients (42%) as compared with those subjects without T2DM (11%) (p < 0.001). A multivariate regression model revealed that fibrosis stage (p = 0.003) but not T2DM (p = 0.151) was a significant predictor of sCD14 levels.

Sub-analysis examined the impact of diabetic status on biochemical factors, which determined that glucose and ALT were significantly different between NAFLD diabetics when compared with NAFLD non-diabetics (glucose: p < 0.0001, ALT: p = 0.024).

### Therapeutic influence of Orlistat on metabolic markers in NAFLD patients

No significant changes in body weight and metabolic markers were observed in six patients treated with diet for one year (Table 3). In contrast, eight patients that also received Orlistat exhibited a significant reduction in body weight post 6 and 12 month treatment (p = 0.001 and p = 0.005, respectively). Furthermore, circulating levels of endotoxin were significantly reduced in Orlistat treated patients (p = 0.012) after one year. With regard to serum ALT, reduced levels were observed in both groups at 6 and 12 months compared with baseline. The reduction at 6 months in Orlistat treated patients was statistically significant (p = 0.017), whilst there were no significant changes in serum levels of sCD14 and lipids. Finally, changes in endotoxin levels did not correlate with changes in any other metabolic parameters.

**Table 3:** Clinical/biochemical characteristics of NAFLD patients on diet and Orlistat treatment

	Diet alone			Diet and Orlistat		
	Baseline	6 months	12 months	Baseline	6 months	12 months
**Age (years)**	48.8 ± 11.5			53.4 ± 16		

**Body weight (kg)**	100.0 ± 16.1	100.8 ± 16.3	101.6 ± 16.3	100.9 ± 24.5	95.5 ± 24.4 *	96.4 ± 25.7 *

**Endotoxin (EU/mL)**	15.9 ± 7.2	16.7 ± 5.5	14.4 ± 11.0	15.8 ± 4.6	14.4 ± 5.7	11.1 ± 4.0 **

**sCD14 (μg/mL)**	1.52 ± 0.40	2.46 ± 0.19	2.25 ± 1.28	1.45 ± 0.60	1.55 ± 0.35	1.59 ± 0.69

**Glucose (mmol/L)**	6.5 ± 2.4	6.1 ± 1.7	8.2 ± 3.8	5.6 ± 0.9	5.3 ± 0.7	5.4 ± 1.4

**Insulin (μU/mL)**	24.2 ± 20.2		21.2 ± 9.5	22.6 ± 13.5		21.6 ± 13.1

**HOMA-IR**	3.2 ± 2.6		3.0 ± 1.3	2.9 ± 1.8		2.8 ± 1.6

**ALT (U/L)**	129 ± 86	90 ± 67	100 ± 68	93 ± 31	60 ± 20 **	66 ± 41

**Cholesterol (mmol/L)**	4.9 ± 0.8	4.8 ± 1.4	4.7 ± 1.1	5.1 ± 1.4	4.6 ± 1.6	4.9 ± 1.7

**LDL-cholesterol (mmol/L)**	2.8 ± 0.8	3.0 ± 1.2	2.8 ± 1.0	3.1 ± 1.3	2.7 ± 1.4	2.8 ± 1.6

**HDL-cholesterol (mmol/L)**	1.09 ± 0.17	1.62 ± 0.58	1.68 ± 1.35	1.16 ± 0.26	1.38 ± 0.39	1.23 ± 0.36

**Triglycerides (mmol/L)**	2.4 ± 1.3	2.3 ± 1.0	2.4 ± 1.6	1.9 ± 0.6	1.7 ± 0.6	1.9 ± 0.7

NAFLD patients underwent a 12 month course of intensive diet-treatment with (n = 8) or without) Orlistat (n = 6). Data are mean (± SD). * p < 0.005 (versus baseline), ** p < 0.05 (versus baseline).

## Discussion

In the present study we have identified that patients with NAFLD are characterised by a significant increase in circulating levels of endotoxin. This finding was independent of diabetic status and, as such, suggests that endotoxin levels may represent an important early marker of potential liver abnormality. The study also observed an increase in serum levels of sCD14 and sTNFRII within the NASH group; both these markers of inflammation increased as liver disease progressed, as determined by fibrotic stage, with clear significance noted co-current with severe stages of fibrosis. Taken together, these findings are indicative of the severity of associated inflammation through the progression of NAFLD.

Specifically, raised levels of endotoxin have been highlighted as a secondary insult in patients with alcoholic liver disease [9] as well as a potential mediator of inflammation in patients with T2DM [13]. Increased levels of endotoxin have been observed in animal models of NAFLD [15,18] and manipulation of the gut flora has been associated with reduced hepatic inflammation, as a direct result [19,20]. However, the role of endotoxinaemia in human NAFLD remains unclear. Prior studies have illustrated that bacterial overgrowth may impact on disease progression, as examined in 22 patients with NAFLD [21]; however, serum levels of TNF-α were twice as high in patients compared with healthy controls, whilst no difference in endotoxin levels between patients and controls was observed. In contrast to these findings, recent studies have shown a five-fold elevation of serum endotoxin levels in 16 patients with NAFLD [22]. This apparent discrepancy may align with the endotoxin assay and how this is performed, as it requires careful technical execution as well as ensuring assay comparison of cohorts is undertaken under the exact same assays conditions, with appropriate validation [13].

Our present studies report the largest studied cohort of NAFLD patients' circulating endotoxin levels. The current findings clearly indicate that endotoxin levels in the peripheral circulation are increased in patients with NAFLD, with no discernible differences between levels in NAFLD and NASH subjects. However, due to the cross-sectional nature of the present study, it was not possible to determine whether increased endotoxin levels are the cause or consequence of NAFLD. Accumulating evidence does indicate that elevated levels of endotoxin may, indeed, play a role in metabolic disease [13,14]. Notably, endotoxin seems to promote liver fibrogenesis by stimulating TLR4, as elegantly shown in three different mouse models of liver fibrosis [23]. In the present study, endotoxin levels were elevated in all fibrosis stages of liver disease, although no clear association between stages and serum endotoxin levels was identified. The sCD14 levels showed a positive trend with disease progression, which was noted as significant at fibrosis stages 2-4, compared with controls, and was also evident by the presence of higher sCD14 levels in NASH compared with NAFLD subjects. However, no association between endotoxin and sCD14 levels was observed, which may be a result of sCD14's duplicitous function. Soluble CD14 is considered to enhance endotoxin clearance from serum [24] whilst also having an active role in endotoxin induced activation of macrophages, as the TLR and sCD14 complex responds to an acute phase response, recruiting further macrophages [8]. Studies by Moreno and colleagues identified that the inhibition of sCD14, via the administration of monoclonal antibodies, in a system absent of membrane bound CD14, blocked monocyte activation [25]. Furthermore, that the introduction of sCD14, present in replacement serum, initiated the LPS/endotoxin response once more [25]. Similarly, in a study by Lloyd *et al*, the application of serum devoid of sCD14 prevented low level detection of LPS, whilst the introduction of recombinant sCD14 restored this response [26]. Therefore, the essential role of sCD14 in the activation of LPS/endotoxin may explain the lack of any association between endotoxin and sCD14 levels in our current findings and, perhaps, the slight decrease in endotoxin levels with increasing liver damage (between fibrosis stage 3 & 4). It should also be noted that, as the serum samples were not pre-heated in this study, the assay measured unbound, accessible endotoxin. As sCD14 complexes with LPS to activate the TLR-4 pathway, the increase in sCD14 levels that occurs with the progression of liver disease might explain the reduction in endotoxin levels, as more is bound to sCD14 and inaccessible for endotoxin detection. Similar findings have previously been reported, with no relationship identified between endotoxin and sCD14 in disease states including malaria and meningococcal septic shock [27,28]. In these studies, the results indicate that sCD14 does not provide a useful early marker for disease detection, which is in accord with our present findings.

No significant difference in endotoxin levels was observed between patients with simple steatosis (NAFLD) and patients with NASH, yet the significant correlation between sTNFRII and sCD14 levels may still reflect the presence of endotoxin induced inflammation in patients with NAFLD. Again, sTNFRII is known to remain elevated for a longer duration than TNFα, thus proving to be a more indicative measure of activity of the TNFα system [17]. As a result, previous studies have noted sTNFRII to correlate with the severity of liver damage - which is confirmed by our present findings [29,30]. Interestingly, TLR4-signaling has recently been shown to play a crucial role in the development of hepatic inflammation in a mouse model of NASH [18]. Further, mice fed on a high fructose diet resulted in the development of NASH co-current with an association with increased endotoxin concentration in portal blood [20]. Moreover, in humans, a mutation in the promoter for CD14, which leads to increased transcriptional activity, is associated with increased susceptibility for NASH [31].

The possible detrimental effects of endotoxin are not necessarily restricted to the liver. It is now widely recognised that insulin resistant states, such as T2DM, cardiovascular disease and the metabolic syndrome, are characterised by a low-grade systemic inflammation, as well as inflammatory changes in adipose tissue [6,13,32,33]. Supporting the role for endotoxin in this context, we have previously reported that endotoxin exerts proinflammatory effects on human adipocytes *in vitro *[13]. Further support for an association between endotoxin and insulin resistance has been identified by Cani *et al *who have shown, using mouse models, that continuous infusion of endotoxin for four weeks induced identical metabolic changes as those induced by a high-fat diet, namely insulin resistance and weight gain [14]. Additionally, the use of CD14 mutant mice caused a reduction to most of the LPS and high-fat diet-induced detrimental changes. As such, the authors suggest that the CD14/LPS system sets the tone for insulin sensitivity. This is in accord with our current findings, as determined by the positive correlations identified between endotoxin and insulin levels, as well as insulin resistance. As insulin resistance is almost universally present in NAFLD, chronic endotoxinaemia may be of particular importance in this condition, not only as a factor that induces hepatic inflammation and fibrosis, but also as a factor contributing to insulin resistance.

The causes of increased blood levels of endotoxin in patients with NAFLD are not clear with several explanations to be considered, such as increased amounts of endotoxin in the intestinal lumen, increased intestinal absorption and reduced clearance from the blood. It is possible, if not likely, that the amount of endotoxin in the intestinal lumen depends on the type of bacteria present, thus, obesity-associated changes in the gut flora, as recently reported in both humans [34] and mice [35], could have important metabolic consequences. Moreover, intestinal dysmotility and/or bacterial overgrowth have been reported in diabetic patients [36], as well as in NASH patients [21,37]. In a recent study by Miele *et al*, NAFLD subjects were shown to have both increased gut permeability and prevalence of small intestinal bacterial overgrowth [12]. In addition, the level of bacterial overgrowth correlated with the severity of steatosis in the NAFLD patients, supporting the theory that such disturbances could possibly facilitate increased absorption of endotoxin from the gut. Interestingly, Brun and co-workers have demonstrated disrupted intestinal tight junctions in a rodent model of the metabolic syndrome, a finding that has also recently been confirmed in NASH patients [12,15], thus providing strong evidence for an anatomical basis underlying increased gut permeability. Furthermore, a susceptibility to gut leakiness has been noted in humans with NAFLD after challenge with aspirin [22]. In a more recent study by Ghoshal and co-workers, a mechanism for the simultaneous absorption of fat and LPS was identified. Long chain dietary fats are incorporated into chylomicrons, which also have a high affinity for LPS and can therefore transfer it from the gut to the bloodstream [11]. Our present and previous findings, in which strong correlations between triglyceride and endotoxin levels are apparent, would support the fat mediated uptake of LPS [32,38]. Such a mechanism might explain the results from this study, which identified that treatment with Orlistat was associated with a significant reduction in endotoxin levels. This effect has also been observed in eighteen subjects with impaired glucose tolerance, all of which were treated with Orlistat for one year [38]. As treatment with Orlistat has previously been associated with beneficial metabolic effects independent of weight loss [39], the current findings suggest that reduced absorption of endotoxin may occur through the blockade of dietary fat absorption, via the mechanism proposed by Goshal *et al*. This hypothesis must, however, be tested in a larger, randomised, controlled trial.

In conclusion, the present study confirms that circulating endotoxin levels are elevated in patients with NAFLD. This result gives further support to the concept that chronic endotoxinaemia could be an important pathogenic factor in NAFLD and that elevated endotoxin levels may serve as an early biomarker for potential liver damage. Studies exploring the impact of the gut flora on human metabolism are now needed to further assess this hypothesis. If the gut flora turns out to be an important determinant of endotoxin levels in humans, treatment with probiotics or lipase inhibitors may prove to be beneficial in metabolic diseases, particularly in NAFLD.

## Competing interests

The authors declare that they have no competing interests.

## Authors' contributions

ALH for the design, statistical analysis, manuscript development and final revision of the paper, NFS for the design, statistical analysis, drafting of the manuscript and manuscript development; SJC for the drafting and revising of the manuscript; KM, TB and EMY for their practical and intellectual input; GT for the statistical analysis and interpretation of data; EA, MSA HMS and AIA for their interpretation of data and intellectual input; ADB for the acquisition and interpretation of data; SK for the interpretation of data and intellectual input; CPD for the concept, acquisition and interpretation of data; PM for the concept, design, interpretation of data and intellectual input. All authors read and approved the final manuscript.
